# Robust neural networks using stochastic resonance neurons

**DOI:** 10.1038/s44172-024-00314-0

**Published:** 2024-11-13

**Authors:** Egor Manuylovich, Diego Argüello Ron, Morteza Kamalian-Kopae, Sergei K. Turitsyn

**Affiliations:** Aston Institute of Photonic Technologies, Aston University, Birmingham, UK

**Keywords:** Computational science, Applied physics

## Abstract

Various successful applications of deep artificial neural networks are effectively facilitated by the possibility to increase the number of layers and neurons in the network at the expense of the growing computational complexity. Increasing computational complexity to improve performance makes hardware implementation more difficult and directly affects both power consumption and the accumulation of signal processing latency, which are critical issues in many applications. Power consumption can be potentially reduced using analog neural networks, the performance of which, however, is limited by noise aggregation. Following the idea of physics-inspired machine learning, we propose here a type of neural network using stochastic resonances as a dynamic nonlinear node and demonstrate the possibility of considerably reducing the number of neurons required for a given prediction accuracy. We also observe that the performance of such neural networks is more robust against the impact of noise in the training data compared to conventional networks.

## Abstract (web summary)

Manuylovich and colleagues propose the use of stochastic resonances in neural networks as dynamic nonlinear nodes. They demonstrate the possibility of reducing the number of neurons for a given prediction accuracy and observe that the performance of such neural networks can be more robust against the impact of noise in the training data compared to the conventional networks.

## Introduction

**Fig. 1:**
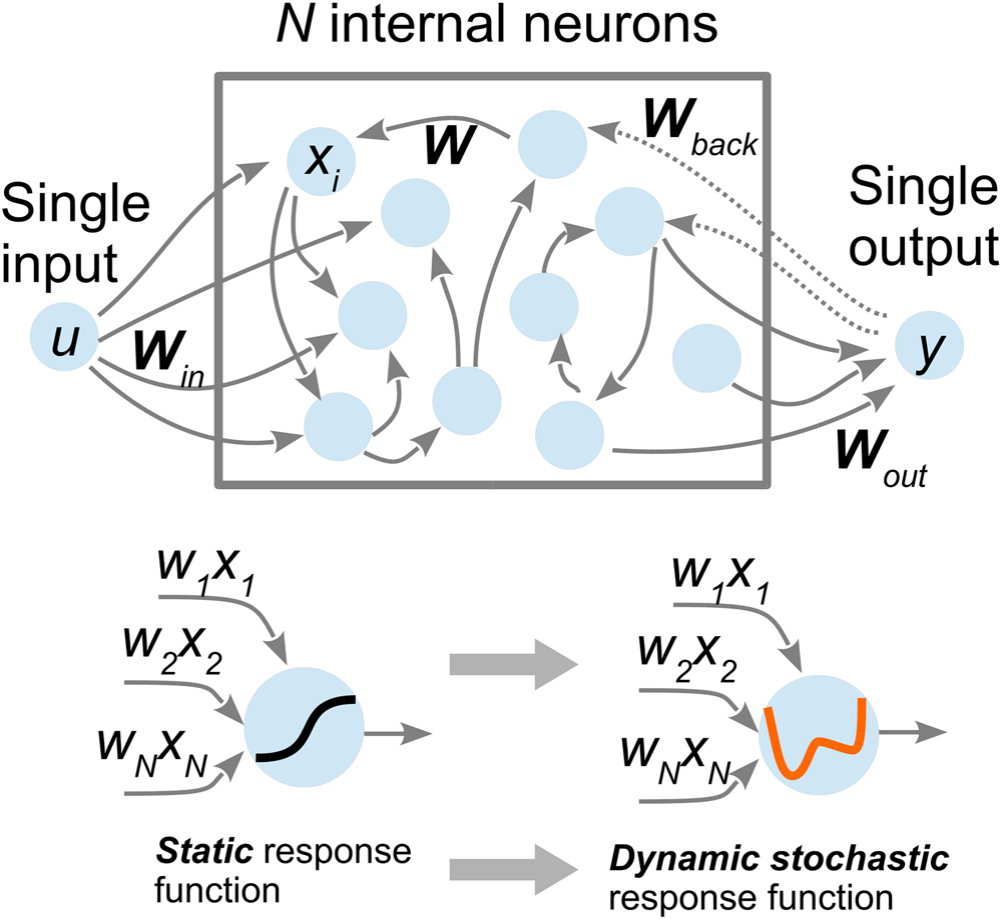
Scheme of the considered echo state network and the proposed use of dynamic stochastic response functions instead of static ones.

Artificial neural networks (ANNs) are capable of solving certain problems using only the accessible sets of training examples, without knowledge of the underlying systems responsible for the generation of this data (see e.g., refs. ^1–3^ and references therein). In scientific and engineering applications, ANNs can be employed as a nonlinear statistical tool that learns low-dimensional representations from complex data and uses this to model nontrivial relationships between inputs and outputs. The ability of ANNs to predict and approximate from given data is linked to the effective dimensionality—the number of independent free parameters in the model. The approximation capability of the ANNs is quantified by the universal approximation theorems (see for details, e.g., ref. ^4^). Note a recent proposal of the Kolmogorov-Arnold Networks^5^ that exploits as the foundation for approximation the Kolmogorov-Arnold representation theorem^6,7^. While traditional deep learning ANNs based on multi-layer perceptron with a fixed activation functions on nodes (“neurons”), KANs have adjustable activation functions on edges (“weights”) and no linear weight matrices at all. However, each weight parameter is replaced by a learnable one-dimensional function parametrized as a spline that can also have high complexity. The complexity of the ANNs plays a critical role in the trade-off between their performance and accuracy of the predictions on the one side and power consumption and speed of operation on the other. Many modern applications of ANNs require over-parametrized models to ensure optimal performance, resulting in high computational complexity and corresponding increased power consumption. Power efficiency can be improved using physically implemented (not necessarily digital) neural networks, for instance, designed from layers of controllable physical elements^8^. Physics-inspired or physics-informed analog neural networks, capable of combining data processing with the knowledge of the underlying physical systems embedded into their architecture^8–12^, is a fascinating area of research offering, in particular, a potential pathway to power-efficient ANNs. There is, however, an essential trade-off between improvements in energy efficiency and susceptibility to noise in analog networks. The fundamental challenge in non-digital systems is the accumulation of noise originating from analog components^13–16^. To overcome this challenge and unlock the full potential of analog ANNs, systems must be developed capable of absorbing, converting, and transforming noise. This paper proposes a new artificial neural network design using a stochastic resonance (SR)^17^ as a network node—ANN-SR. The robustness of the ANNs operation in the presence of noise depends on the properties of the nonlinear activation function^16^. Instead of using a conventional static nonlinear element, we employ a dynamical system with bi-stable features (see Fig. 1) that can make a positive use of the noise—the stochastic resonance (SR). Note that various experimental implementations of SRs pave the way to new attractive, physically implementable designs of ANNs^18,19^. SR is a phenomenon that occurs when adding noise to a nonlinear system can improve the system’s performance. It is often observed in physical systems, for example, in electronic^18,20,21^, mechanical^22,23^, or even quantum^24^ systems. SR has also been observed in a wide range of biological systems, including neurons^25^, cells^26^, and even hearing^27^. Note that the idea of using SR as a nonlinear activation function in ANNs has been studied^28–30^. However, these works did not address the key potential advantage of SR - its ability to make under certain condition a positive use of noise.

Here we demonstrate that SR being employed as a nonlinear activation function can notably improve the performance of ANNs when trained on noisy data. We also show that use of SR activation function can substantially reduce the computational complexity and the required number of neurons for a given prediction accuracy. Our work uniquely demonstrates the robustness of SR neurons when trained with noisy datasets, showing improved prediction accuracy compared to traditional sigmoid functions. This application is particularly relevant for real-world scenarios where data is often imperfect. Additionally, we have performed a detailed computational complexity analysis, showing that while our SR-based approach is marginally more computationally expensive than classical approaches, it offers superior performance benefits. This trade-off is crucial for practical implementations where both accuracy and computational resources are considered. An error of 10^−2.76^ or 0.0017 was achieved for predicting 100 points of the Mackey-Glass series by using only 40800 multiplications per point. For the classical sigmoid activation function, an error of 10^−1.45^ or 0.036 corresponds to a similar number of multiplications. This means that we obtain 20 times more accurate results by using the same number of multiplications. To achieve the same accuracy using the sigmoid function, 203850 multiplications are needed. Thus, to achieve the same accuracy as using the classical approach, one needs to perform five times more multiplications and spend five times more energy. Moreover, ANN-SR performance is more robust against noise in the case of training on noisy data.

## Results

As an illustration of our general idea, we consider a particular recurrent neural network schematically shown in Fig. 1, known as echo state network (ESN)^31^. However, the idea of SR neurons can be easily extended to other architectures, such as e.g., deep neural networks. ESNs are fixed recurrent neural networks that constitute a “reservoir” with multiple (fixed) internal interconnections providing a complex nonlinear multidimensional response to an input signal. An output signal is obtained by training linear combinations only of these readout ESN responses. The ESN has an internal dynamic memory. The internal connections of the ESN are randomly set up. During the training procedure, the training sequence is fed into the ESN. After an initial transient period, the neurons start showing variations of the fed signal or “echoing” it. The readout weights are calculated after feeding the training sequence to the ESN.

To explain the mathematics behind our idea, we consider the classical ESN model^31^:

1 $${x}_{n+1}=f({{{\bf{W}}}}_{{{\rm{in}}}}{u}_{n+1}+{{\bf{W}}}{x}_{n}+{{{\bf{W}}}}_{{{\rm{back}}}}\,{y}_{n})$$

where $u\in {\mathbb{R}}$ is the current state of the ESN’s input neuron, ${x}_{n}\in {{\mathbb{R}}}^{N\times 1}$ is the vector with dimension *N* corresponding to the network’s internal state, matrix ${{{\bf{W}}}}_{{{\rm{in}}}}\in {{\mathbb{R}}}^{N\times 1}$ is the map of the input *u* to the vector of dimension *N*, the matrix ${{\bf{W}}}\in {{\mathbb{R}}}^{N\times N}$ is the map of the previous state of the recurrent layer, and *f*(*x*) is the nonlinear activation function. The recurrent layer consists of *N* neurons and the input information *u* contains a single element at each instance of time. The linear readout layer is defined as

2 $${y}_{n}={{{\bf{W}}}}_{{{\rm{out}}}}{x}_{n}$$

where vector ${{{\bf{W}}}}_{{{\rm{out}}}}\in {{\mathbb{R}}}^{1\times N}$ maps the internal state of the ESN to a single output.

In the classical approach, the nonlinear activation function *f*(*x*) is typically a sigmoid or hyperbolic tangent function^31^. In our model, we replace the analytical nonlinear function *f*(*x*) with a stochastic ordinary differential equation known as stochastic resonance (SR)^32,33^. The SR phenomenon has been studied in various physical systems, including climate modeling, electronic circuits, neural models, chemical reactions, and photonic systems^17,32–35^. This dynamic can be described using a bi-stable system with two inputs: a coherent signal and noise^34,36^. A standard example of the SR model is given by:

3 $$\frac{d\xi (t)}{dt}=-\frac{d{U}_{0}(\xi )}{d\xi }+s(t)+\sigma {{\mathcal{N}}}(t)$$

where *s*(*t*) represents the input signal, ${{\mathcal{N}}}(t)$ is Gaussian noise with zero mean and unit variance, and *σ* is the noise amplitude. The potential *U*(*ξ*, *t*) is defined as:

4 $$U(\xi ,t)={U}_{0}(\xi )+\xi s(t)$$

Considering a symmetric bi-stable stationary potential well *U*_0_:

5 $${U}_{0}(\xi )=-\alpha \frac{{\xi }^{2}}{2}+\beta \frac{{\xi }^{4}}{4}$$

This model represents the limit of a heavily damped harmonic oscillator, depicting the movement of a particle in a time-dependent bi-stable potential *U*(*x*, *t*) = *U*_0_(*x*) − *xs*(*t*)^36,37^. For *α* > 0, the potential is bi-stable with two stationary points ${x}_{s}=\pm \sqrt{\alpha /\beta }$ and a barrier Δ*U* = *α*^2^/(4*β*). In this work, we consider the SR function with the potential function with local minima of −1 and 1. Therefore, we assume, without loss of generality, *α* = *β* The time-dependent tilted potential *U*(*ξ*, *t*) is defined by the static potential function *U*_0_ combined with the time-dependent input signal *s*(*t*), resulting in:

6 $$U(\xi ,t)=-\alpha \left(\frac{{\xi }^{2}}{2}-\frac{{\xi }^{4}}{4}\right)+\xi s(t)$$

We demonstrate that the SR function, under certain conditions, outperforms the classical sigmoid as a nonlinear activation function in terms of prediction accuracy and computational complexity. The proposed SR neuron differs from a standard neuron by having its own internal state *ξ*(*t*), which acts as memory. This state, along with incoming signals, forms the neuron’s output through integration over a time interval Δ*t*.

To evaluate the performance of ANNs with SR activation function, we applied it to the prediction of two-time series for two classical cases: (i) the Mackey-Glass (MG) equation, and (ii) the Rössler attractor.

For the Mackey-Glass series, governed by:

7 $$\frac{dq(t)}{dt}=\frac{aq(t-\tau )}{1+{q}^{10}(t-\tau )}-bq(t)$$

with parameters *a* = 0.2, *b* = 0.1, and *τ* = 17, we also considered a noisy version of the MG series, ${q}_{\eta }(t)=q(t)+\eta {{\mathcal{N}}}(t)$, to assess the signal-to-noise ratio (SNR):

8 $${{\mbox{SNR}}}_{{{\rm{MG}}}}=\frac{\max (q)-\min (q)}{2\eta }$$

For the Rössler attractor, governed by:

9 $$\frac{dx}{dt}=-y-z$$

10 $$\frac{dy}{dt}=x+ay$$

11 $$\frac{dz}{dt}=b+z(x-c)$$

with parameters *a* = 0.2, *b* = 0.2, and *c* = 5.7, we similarly considered a noisy version of the Rössler series to evaluate its SNR:

12 $${{\mbox{SNR}}}_{{{\rm{R}}}\ddot{{{\rm{o}}}}{{\rm{ssler}}}}=\frac{\max (x)-\min (x)}{2\eta }$$

We used the *x*-component of the Rössler attractor to test the proposed SR-ANN performance.

Parts of both the MG series and the Rössler attractor series were used to train the ESN, and the next several tens of points of each series were compared to the output of the freely running ESN to estimate the prediction accuracy.

During the training and testing, the nonlinear response of each SR neuron is calculated as a result of the integration of Eq. (3) with a weighted sum of input neurons as the variable *s* in Eq. (3) and previous internal state of the neuron as an initial condition. As the training and test sequences are parts of the time series (MG or Rössler), they are time-dependent. Therefore, we integrate Eq. (3) over time with *s* as a parameter. The integration interval Δ*t* is equal to the time step of the time series, and we set the initial value of the SR function as $\xi (t=0)={{\mathcal{N}}}(0,1)$. On each time step, we take the current value of the function *ξ*_*n*_ and calculate the next value by integrating Eq. (3) with *ξ*_*n*_ as an initial value:

13 $$f({s}_{n})\equiv {\xi }_{n+1}={\xi }_{n}+\left[\alpha \left({\xi }_{n}-{\xi }_{n}^{3}\right)+\sigma {{\mathcal{N}}}(t)\right]\cdot \Delta t+{s}_{n}\cdot \Delta t$$

This is done whenever we need to calculate the nonlinear response function *f*(*s*) using a 2nd-order Runge-Kutta also known as the Euler method.

The output of the nonlinear response function is calculated as a trajectory of a system described by the SR equation with input values as an external force applied to the system. The internal state of the SR system is described by a vector *ξ* that evolves with time. For a given initial state *ξ*_*n*_ and input value *s*_*n*_, the output of the SR nonlinear function can be calculated as the next internal state *ξ*_*n*+1_ separated from the previous state by time Δ*t*. Note that there are two sources of noise: (i) implementation noise coming from the active nature of the nonlinear node and (ii) the noisy data, both practically important and considered in this work.

In the current work, we use the proposed approach to design an ESN and estimate its accuracy depending on the number of neurons and noise amplitude in the training data. We compare the designed ESN-SR with the classical ESN with sigmoid activation function in terms of accuracy and computational complexity.

To investigate the computational complexity of the proposed approach, we calculate the number of multiplications required for a single step of ESN evolution. This number includes both the linear part of the ESN evolution and the multiplications needed for calculating the nonlinear response of the neurons.

According to equations (1) and (2), the linear part of ESN evolution includes 4 matrix multiplications of size *N* × 1, *N* × *N*, *N* × 1, and 1 × *N* giving a total of

14 $${Q}_{{{\rm{linear}}}}={N}^{2}+3N$$

multiplications per 1 step of the linear part of the ESN evolution. This is also the total number of multiplications for the classical approach for the nonlinear activation function, such as sigmoid or tanh, obtained using a lookup table with no computational complexity.

On the other hand, for the SR nonlinear function, the values of $\alpha (\xi -{\xi }^{3})+\sigma {{\mathcal{N}}}(t)$ or $\left[\alpha ({\xi }_{n}-{\xi }_{n}^{3})+\sigma {{\mathcal{N}}}(t)\right]\cdot \Delta t$ can also be calculated using look-up tables and bear no computational burden. However, *s*_*n*_ ⋅ Δ*t* needs to be calculated and add up *ξ*_*n*_, $\left[\alpha ({\xi }_{n}-{\xi }_{n}^{3})+\sigma {{\mathcal{N}}}(t)\right]\cdot \Delta t$, and *s*_*n*_ ⋅ Δ*t*. So an additional

15 $${Q}_{{{\rm{nonlinear}}}}^{SR}=N$$

multiplications are needed to calculate the SR nonlinear response function.

To sum up, the total numbers of multiplications for 1 step of calculating the evolution of the ESN are:

16 $$\begin{array}{rcl}&&{Q}_{{{\rm{total}}}}^{{{\rm{classical}}}}={N}^{2}+3N\\ &&{Q}_{{{\rm{total}}}}^{SR}={N}^{2}+4N\end{array}$$

Note that the number of additional multiplications grows linearly with the size of the ESN, and the total number of multiplications grows quadratically and *N* > > 1, so the quadratic term dominates in the complexity.

The prediction accuracy is measured by the mean squared error between freely running ESN and the corresponding 100 samples of the time series (MG or Rössler). The parameters of the ESN under test are as below:

- *N* = 50 ⋯ 1000
- Connectivity of **W** is 0.01
- Hyperparameter of the SR function *α* = 0.01
- SR noise amplitude *σ* = 0, 10^−10^, 10^−8^, 10^−6^

**Fig. 2:**
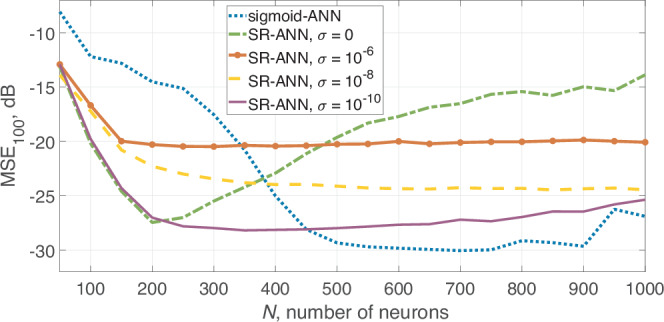
Mean squared error (MSE) for artificial neural networks (ANNs) employing sigmoid (sigmoid-ANN) or stochastic resonance (SR-ANN) nonlinear activation functions and depending on the number of neurons.

The result of this test is shown in Fig. 2, where each point represents a mean value averaged over 1000 samples.

The “classical” ESN with sigmoid activation function is trained under similar conditions. The linear regression problem for determining the readout weights was performed using singular value matrix decomposition.

**Fig. 3:**
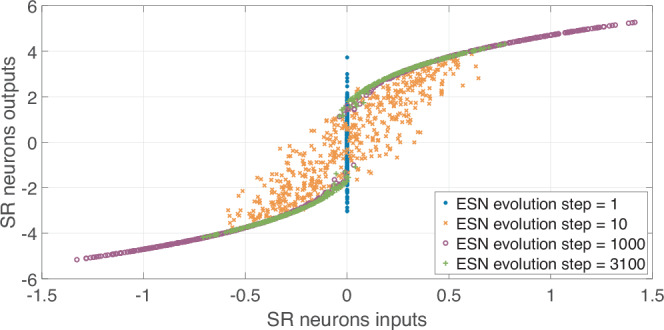
Stochastic resonance transfer function for different moments of the echo state network (ESN) evolution.

The transfer function of an SR neuron depends on the number of ESN evolution steps. The initial values (step 1) are normally distributed with mean 0 and variance 1. The transfer function for steps 1, 10, 1000, and 3100 are shown in Fig. 3. Each neuron automatically converges into its transfer function during the training procedure, allowing self-adjusting activation functions in different parts of ESN using the same node design. For the lower number of neurons, the performance of ESN-SR is better than the classical approach with a sigmoid activation function. In particular, the SR method reaches its maximum accuracy at *N* = 200 neurons with an averaged error of 10^−2.76^. The number of multiplications per 1 step is ${Q}_{{{\rm{total}}}}^{SR}(N=200)=40800$. For the classical sigmoid an error of 10^−1.45^ or 0.036 is achieved for a similar number of nodes ${Q}_{{{\rm{total}}}}^{{{\rm{classical}}}}(N=200)=40600$. So we obtain 20 times more accurate results by using the ESN of the same computational complexity. To achieve the same accuracy using the sigmoid activation function, one needs to take *N* = 450 neurons leading to ${Q}_{{{\rm{total}}}}^{{{\rm{classical}}}}(N=450)=203850$ multiplications per 1 step. So, to achieve the same accuracy using the classical sigmoid function, one needs to perform 5 times more multiplications and 2.5 times more nodes.

It should be mentioned that the maximum accuracy achieved by using the sigmoid function is slightly higher, but at the expense of much more computational cost: the maximum accuracy of 10^−3^ is achieved at *N* = 700 neurons that require ${Q}_{{{\rm{total}}}}^{{{\rm{sigmoid}}}}(N=700)=492100$ multiplications per 1 step. So the best result is only 1.75 times better compared to the best SR result, while it is achieved by using 12 times more multiplications.

**Fig. 4:**
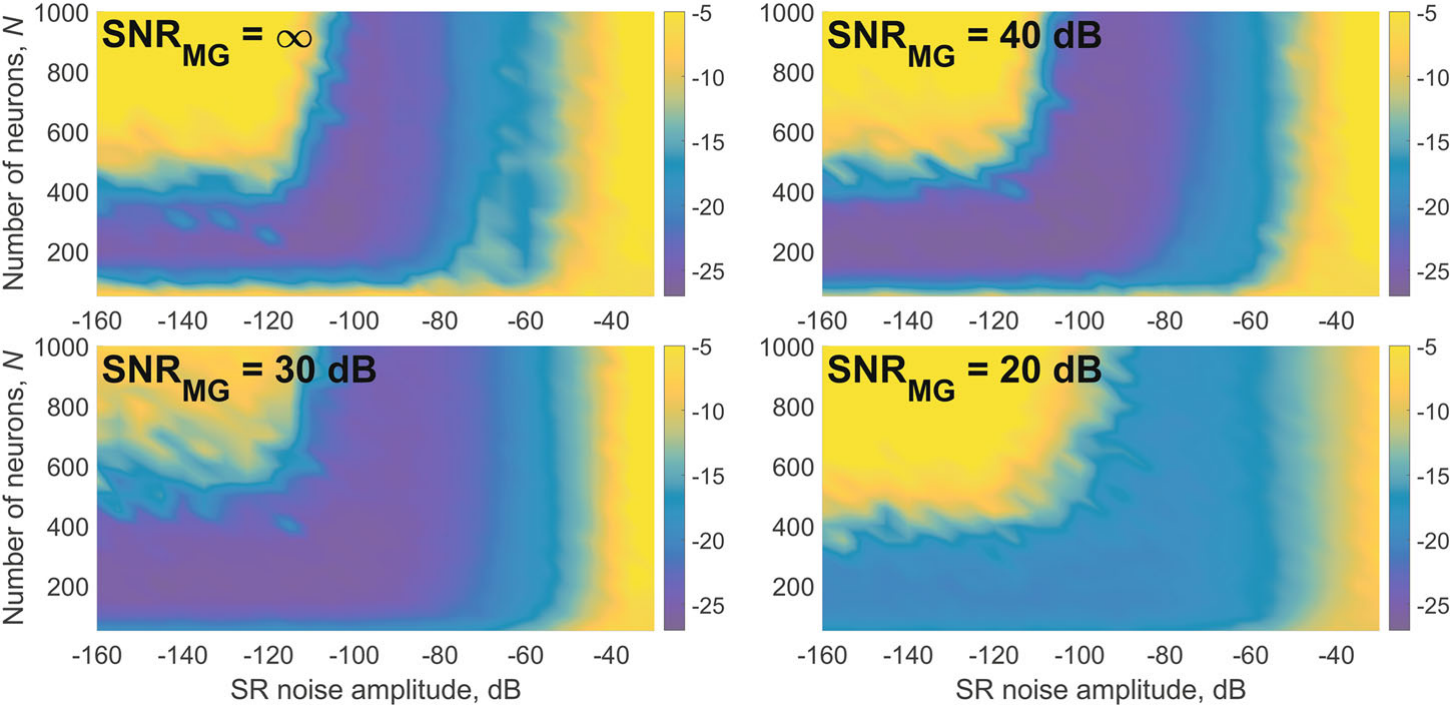
Prediction accuracy *MSE*_100_, dB (color-coded) depending on stochastic resonance noise level, number of neurons, and noise in the training sequence.

We investigated how the noise amplitude *σ* affects the accuracy and stability of the ESN, see Figs. 2 and 4. One can see that the low level of added noise slightly improves the maximum accuracy and increases stability and accuracy at a higher number of neurons by preventing overfitting. But even slightly higher noise amplitude reduces the accuracy. We also investigated the capability of predicting the continuation of the time series (MG or Rössler) when training on noisy sequences. The training sequence was corrupted by white Gaussian noise and fed into the ESN with the same training procedure.

The dependence of the MSE (color-coded) on the number of neurons, internal noise in the nonlinear activation function, and SNR of the training sequence is shown in Fig. 4 showing the optimal internal SR noise level of 10^−10^ for SNR_MG_ = *∞*, level of 10^−9.5^ for SNR_MG_ = 40 dB, level of 10^−9^ for SNR_MG_ = 30 dB and level of 10^−8^ for SNR_MG_ = 20dB.

**Fig. 5:**
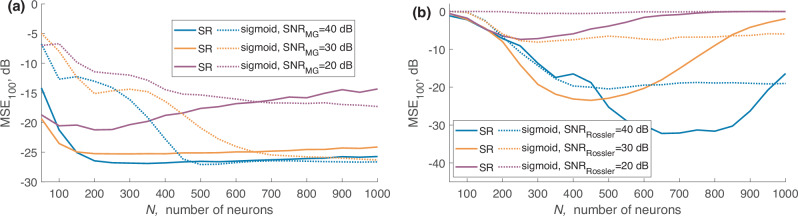
Accuracy of the reservoir computer on noisy time series. Accuracy of the reservoir computer when training on (**a**) noisy Mackey-Glass (MG) and (**b** noisy Rössler sequences with various signal-to-noise ratios (SNR) in the training sequences. Solid lines represent performance using stochastic resonance (SR), while dashed lines correspond to the standard sigmoid function.

We used the SR function with *σ* = 10^−10^ and compared it with the classical sigmoid function. Noise amplitudes in the training sequence *σ* corresponding to SNR of 20, 30, and 40 dB were chosen. Figure 5 shows how the MSE of the first 100 predicted values depends on the number of neurons for different nonlinear activation functions and various noise levels in the training sequence.

**Fig. 6:**
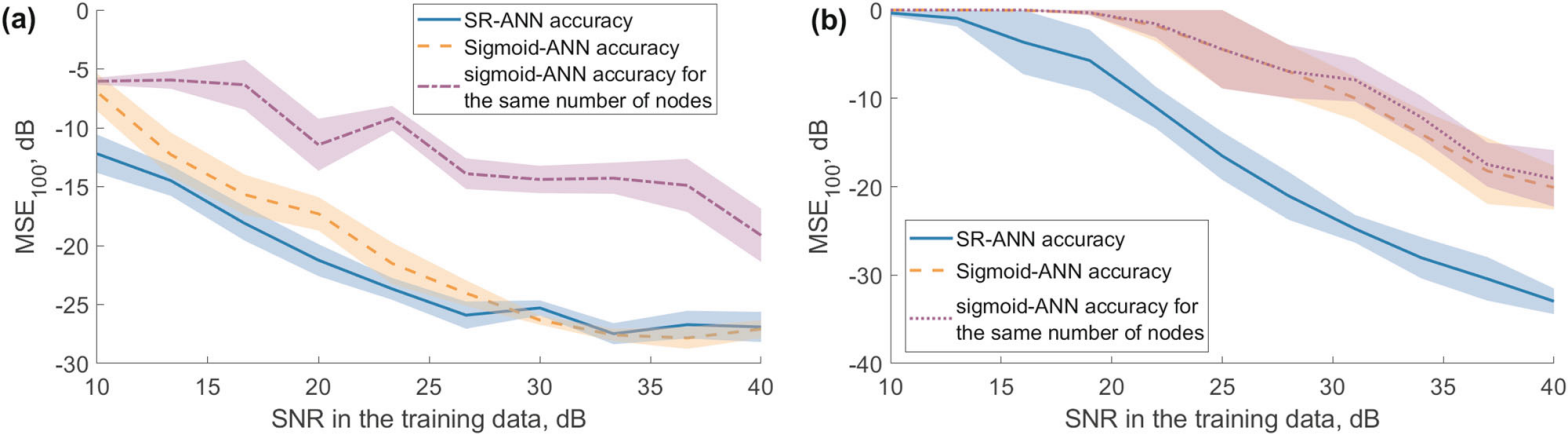
Comparison of accuracy across ANNs with different nonlinear functions. Best accuracy across artificial neural networks (ANNs) of varying complexity, comparing sigmoid-ANN and stochastic resonance ANN (SR-ANN) nonlinear functions on (**a**) Mackey-Glass series and (**b**) Rössler attractor series. Shaded areas depict one standard deviation interval.

The proposed method shows superior performance compared to the classical approach in the case of the lower number of neurons and the same performance for the higher number of neurons. In particular, in the case of SNR = 20 dB, the prediction accuracy is as good as 0.01 when the number of neurons is as low as 100 in the case of SR. The use of the sigmoid function provides 24 times less accurate results at this number of neurons. And this accuracy is never achieved with the classical sigmoid function, even at a higher number of neurons at this noise level in the training sequence. Figure 6 shows how the best prediction accuracy across various numbers of neurons depends on the SNR in the training sequence where shaded regions depict one standard deviation interval calculated on 1000 runs.

As can be seen in Fig. 6a, while there is no statistically significant difference between the performance of the ESN-SR and sigmoid systems on MG for SNR > 25 dB, the latter outperforms the former in other regions, especially when the number of nodes is the same (violet curve).

In the context of Rössler attractor series, as depicted in Fig. 6b, the performance of the SR-ANN method remains consistently superior. Specifically, for SNR values below 25 dB, the SR-based approach exhibits notably better accuracy compared to the sigmoid-based approaches, irrespective of the number of neurons used. These results highlight the robustness of the SR-ANN method. This is particularly important for training on experimental data, which is often subject to noise, suggesting that SR-ANN might offer more reliable performance in practical scenarios where noise is prevalent.

## Discussion

On a general level, we would like to note that our approach is different from the designing network through combining simple elements defined in the corresponding mathematical theory. Instead, we build computing system using the existing building block based on (relatively simple) physical system. In this particular example, we used stochastic resonance, however, this rather general approach can be applied to various physical systems and sub-systems. We have shown that replacing the standard nonlinear function with SR considerably increased the accuracy of the ESN with fewer neurons when trained on a Mackey-Glass series. The proposed approach requires two times fewer neurons than the classical one to achieve an error of 0.0015: the computational complexity, in this case, has decreased by a factor of 4. Moreover, since noise is ubiquitous in any real implementation of an ESN, a more realistic investigation requires considering the impact of noise introduced to the system through the nonlinear activation node. Our simulations show that ESN-SR outperforms the one with conventional nodes by a margin that is dependent on the noise power. With a low noise level, the accuracy of the ESN is slightly improved, and overfitting is notably reduced for a larger number of neurons. When training on noisy data, the proposed approach is considerably superior to the classical one in both accuracy and computational complexity. When SNR = 40 dBm and with the same computational complexity, ESN-SR achieves a 25x accuracy improvement compared to the ESN-sigmoid. The classical approach requires more than four times more calculations to deliver the same accuracy. This difference becomes more prominent for noisier data: for SNR = 20 dB with the same computational complexity, the best accuracy of the proposed method achieved is 9.5 times better than the one with sigmoid nodes.

This indicates the capability of SR nodes to capture the underlying relations between samples of the input and manifesting memory properties in various ANNs.

SR-based neurons perform better in certain scenarios due to the unique properties of stochastic resonance that are leveraged in our network design. SR is a phenomenon where adding noise to a nonlinear system enhances its performance. This counterintuitive effect allows SR-based neurons to make positive use of noise, thus improving overall network performance. In traditional neural networks, performance of static nonlinear activation functions is often negatively affected by noise in the data. In contrast, SR-based neurons employ a dynamical system with bi-stable features that make positive use of noise, converting it into a beneficial element rather than a detrimental one. This is particularly advantageous in real-world applications where data is often imperfect and noisy.

We believe that the proposed idea of using a model (or, indeed, physical systems) governed by stochastic ordinary differential equations can be applied to a range of ANNs and can be generalized to different tasks. In particular, the proposed concept is compatible with high-bandwidth optical analog ANNs and reservoirs, offering potential solutions for high-speed parallel signal processing and reduction in power consumption in physical implementations.

## Methods

We used the classical training procedure to train the designed ESN as described in ref. ^31^. This procedure is as follows:

1. Feed the first 3000 elements of the time series (MG or Rössler) into the output neuron of the ESN. The feeding is the following procedure:
  17 $$\begin{array}{rcl}&&{y}_{n+1}={q}_{n+1}\\ &&{x}_{n+1}=f({{\bf{W}}}{x}_{n}+{{{\bf{W}}}}_{back}{y}_{n})\end{array}$$
  During this, the ESN must start “echoing” the fed signal: after some transitional period, the values of internal weights *x* start to show some variations of the fed signal.
2. Use the last 2000 internal states to train the output weights so at each step, the product of the output weights matrix and the internal state vector gives the corresponding output value:
  18 $${{{\bf{W}}}}_{out}=\arg \min _{{{{\bf{W}}}}_{out}}\sum | {{\bf{X}}}{{{\bf{W}}}}_{out}-y{| }^{2}$$
  Here **X** is a matrix composed of the last 2000 internal states *x*. **W**_*out*_ can be determined by finding the minimum norm least-squares solution to the linear equation.

Then the training sequence is disconnected from the ESN, and the ESN runs freely. The next several output values the freely running ESN produces are compared to the true values produced by the MG equation.

## Data Availability

The data that supports the findings of this study is available from the corresponding author upon reasonable request.

## Code availability

Code for implementing the SR neurons, ESN, and its training and validation algorithms are available from the corresponding author upon reasonable request.
